# Genetic matching for time-dependent treatments: a longitudinal extension and simulation study

**DOI:** 10.1186/s12874-023-01995-5

**Published:** 2023-08-09

**Authors:** Deirdre Weymann, Brandon Chan, Dean A. Regier

**Affiliations:** 1 Cancer Control Research, BC Cancer, Vancouver, Canada; 2 School of Population and Public Health, University of British Columbia, Vancouver, Canada

**Keywords:** Longitudinal matching, Time-dependent treatment, Propensity score, Monte Carlo simulation, Machine learning

## Abstract

**Background:**

Longitudinal matching can mitigate confounding in observational, real-world studies of time-dependent treatments. To date, these methods have required iterative, manual re-specifications to achieve covariate balance. We propose a longitudinal extension of genetic matching, a machine learning approach that automates balancing of covariate histories. We examine performance by comparing the proposed extension against baseline propensity score matching and time-dependent propensity score matching.

**Methods:**

To evaluate comparative performance, we developed a Monte Carlo simulation framework that reflects a static treatment assigned at multiple time points. Data generation considers a treatment assignment model, a continuous outcome model, and underlying covariates. In simulation, we generated 1,000 datasets, each consisting of 1,000 subjects, and applied: (1) nearest neighbour matching on time-invariant, baseline propensity scores; (2) sequential risk set matching on time-dependent propensity scores; and (3) longitudinal genetic matching on time-dependent covariates. To measure comparative performance, we estimated covariate balance, efficiency, bias, and root mean squared error (RMSE) of treatment effect estimates. In scenario analysis, we varied underlying assumptions for assumed covariate distributions, correlations, treatment assignment models, and outcome models.

**Results:**

In all scenarios, baseline propensity score matching resulted in biased effect estimation in the presence of time-dependent confounding, with mean bias ranging from 29.7% to 37.2%. In contrast, time-dependent propensity score matching and longitudinal genetic matching achieved stronger covariate balance and yielded less biased estimation, with mean bias ranging from 0.7% to 13.7%. Across scenarios, longitudinal genetic matching achieved similar or better performance than time-dependent propensity score matching without requiring manual re-specifications or normality of covariates.

**Conclusions:**

While the most appropriate longitudinal method will depend on research questions and underlying data patterns, our study can help guide these decisions. Simulation results demonstrate the validity of our longitudinal genetic matching approach for supporting future real-world assessments of treatments accessible at multiple time points.

**Supplementary Information:**

The online version contains supplementary material available at 10.1186/s12874-023-01995-5.

## Background

Quasi-experimental matching methods support causal inference of outcomes in real-world analyses of healthcare programs and technologies [1]. In the absence of a randomized counterfactual, matching can identify an appropriate comparator for treatment, reduce the sensitivity of effect estimates to final model specifications, and yield unbiased effect estimates provided that the ignorability condition is met [2]. Given that unobserved differences remaining across matched treatment groups signal a deviation from ignorability and threaten the validity of causal inference, researchers generally recommend maximizing covariate balance without limit [3]. The most common matching application estimates propensity scores using baseline covariate values [4], which is unable to address time-dependent confounding.

When treatments are accessible at multiple time points, patient eligibility and probability of receiving treatment can change continuously. For example, patients at risk of cardiac arrest may not immediately access a preventative intervention [5, 6]. In oncology, patients diagnosed with cancer often wait to access genomic tests and targeted cancer treatments [7, 8]. Unaccounted variability in time-dependent covariates during these waiting periods can introduce confounding, threatening the validity of comparative analyses. Longitudinal matching methods can account for time-dependent confounding in real-world evaluations of static treatments whose assignment varies over time. Several longitudinal matching methods exist, each aiming to balance covariate histories through matching patients over their longitudinal trajectories [5]. The comparative performance of these methods is poorly established, owing to a lack of simulation frameworks for longitudinal matching. Further, existing methods require iterative, manual re-specifications to balance covariate histories and achieve ignorability required for causal real-world evidence generation.

For time-invariant analyses, genetic algorithm-based matching automates the process of maximizing covariate balance [9]. Through the use of an evolutionary genetic search algorithm, this method iteratively estimates similarity and selects matched controls that minimize baseline group differences according to pre-specified optimization criteria [10]. To our knowledge, genetic matching, and alternative machine learning approaches, have yet to be considered for automating balancing of time-dependent covariate histories. In this study, we propose a novel longitudinal extension for genetic matching to support real-world comparative evaluations. We then develop a simulation framework and evaluate performance of our proposed approach compared to alternative methods, in terms of ability to balance covariates and efficiently estimate unbiased treatment effects. To mimic real-world confounding, we consider both continuous and binary covariates whose values do and do not change over time, exploring sensitivity to distributional and correlation assumptions.

### Longitudinal matching overview

When a randomized comparator for treatment is unavailable, matching methods seek to mirror a randomized study design and identify a control group to infer comparative effects by drawing on observational data. Controls are selected based on the similarity between their observable characteristics and the characteristics of patients who received treatment. When treatment assignment occurs at a single time point and treatment is static, matching considers only baseline, time-invariant covariates for treated patients and eligible controls [5]. If instead, treatment assignment occurs over a longitudinal period, patients’ covariate histories measured from baseline up until the relevant time scale can be considered. Longitudinal matching enables consideration of time-dependent covariates and their histories through creation of a series of pseudo experiments in which some patients are treated and other eligible at-risk patients are not [5]. Each pseudo experiment initiates at time point *t*_,_ when at least one subject receives a treatment. Treated patients are then matched to available controls, based on eligibility, risk status, and covariate histories at *t*.

There are a large number of matching methods available, each varying in terms of relevant time scales, how covariates are adjusted for, measures used to define similarity, and algorithms employed to select matched controls [4, 5]. In the following section, we present three matching approaches to be assessed in simulation, including a novel proposed machine learning-based method for longitudinally matching patients. We consider applicability of each approach for evaluating a static treatment whose assignment varies over time.

#### Nearest neighbour matching on time-invariant propensity scores (Rosembaum and Rubin 1983) [11]

Propensity score matching on baseline covariates is among the most common matching methods and performs well for cross-sectional treatment evaluations [4]. In contrast to exact matching on covariate values, propensity score matching summarizes information on multiple covariates in a single measure, reducing dimensionality and improving ability to select matches. Propensity score matching begins with estimating patients’ conditional probabilities of exposure given covariates, termed propensity scores, using a regression model of the probability of treatment, defined as:

1 $$\mathrm{Pr}\left({z}_{i}=1|x\right)=f({x}_{i}\beta_{k} +{\varepsilon }_{i})$$

where *z*_*i*_ denotes treatment status for individual *i*, *x*_*i*_ are time-invariant covariates observed at baseline and hypothesized to correlate with both the propensity score and the outcome of interest, *β*_*k*_ are corresponding coefficient estimates, *ε*_*i*_ is an error term, and *f* is a link function enabling non-linearity. While many link functions are possible, the logit link is common when estimating propensity scores:

2 $$f\left(s\right)=ln(\frac{s}{1-s})$$

Coefficients for the propensity score model may be estimated through maximum likelihood and are used to determine individual-level propensity scores. After estimating propensity scores, controls are matched to treated patients using a pre-defined matching algorithm and ratio. For example, 1:1 nearest neighbour matching selects one control whose propensity score is closest to that of a treated patient. Matching on the propensity score, or any function of the same covariates, will balance the distribution of those covariates [11, 12].

After matching, balance of entire covariate distributions must be assessed. Any remaining imbalance can signal a deviation from the ignorability condition [3]. To avoid biasing effect estimates, the propensity score model or the matching algorithm must be re-specified until balance is achieved. This iterative and time-consuming process does not guarantee maximization of covariate balance, which has driven the recent emergence of machine learning-based methods automating the process of maximizing balance of baseline covariates [9, 13, 14].

Time-invariant matching methods are frequently used in real-world studies to evaluate time-dependent treatments [15], but these evaluations require a naïve assumption. Researchers must assume comparability between ever treated and never treated patients and only baseline values for time-dependent covariates factor into matching [5]. By ignoring known variability in factors likely to affect patients’ probability of treatment over the period, this approach incorrectly assumes that never treated patients are directly comparable to ever treated patients throughout the entire study period. Failing to account for this longitudinal variation can lead to bias, including immortal time bias, in which treated patients are guaranteed to be outcome-free from baseline until their treatment date and thus have improved relative outcomes [16]. The relative performance of time-invariant matching compared to longitudinal matching in these instances is poorly characterized.

#### Sequential risk set matching on time-dependent propensity scores (Lu 2005) [17]

Risk set matching is the earliest statistical approach for longitudinal matching and involves matching treated patients with untreated patients in the same risk set at a given time point [5, 18]. Lu (2005) [17] extended risk set matching to reduce covariate dimensionality through consideration of a time-dependent propensity score and proposed two matching algorithms: simultaneous or sequential. We focus on sequential risk set matching on a time-dependent propensity score, which Lu (2005) [17] demonstrated performs similarly to simultaneous risk set matching and involves less restrictive assumptions for covariate exogeneity. This approach is common in applied real-world evaluations of treatments whose assignment varies over time [6, 15, 19, 20].

Sequential risk set matching sequentially matches treated patients to not yet treated patients with similar covariate histories up until a given time point. Coarsening of time is determined based on treatment dates. Unlike matching on time-invariant propensity scores, where all patients are eligible for matching at baseline and thus in the same risk set, sequential matching evaluates multiple risk sets over the period for which eligibility is non-constant. At time *t*, patients eligible for matching are included in the same risk set, *R*_*t*_, and are not yet treated at *t-e* for any *e* > 0. Propensity scores are estimated at time *t* using time-to-event regression models. Lu (2005) [17] recommends a Cox proportional hazards model to estimate time-dependent propensity scores:

3 $${h}_{i}\left(t\right)= {h}_{0}\left(t\right)\mathrm{exp}\,({{\beta }_{k}}{\prime}{x}_{i}(t))$$

where Lu (2005) [17] defines the propensity score at any time point as the hazard function. Given that the hazard function is not a probability and its values may exceed 1, it is not technically a propensity score. We therefore consider the hazard function to be a proxied time-dependent propensity score, rather than a true propensity score throughout the following sections.

Following time-dependent propensity score estimation, patients in *R*_*t*_ with similar proxied propensity scores at time *t* are then matched on the following distance:

4 $$Distance\left({x}_{it},{x}_{jt}\right)={{\beta }_{k}}{\prime}{x}_{i}(t)-{{\beta }_{k}}{\prime}{x}_{j}(t)$$

Covariate balance at time of treatment is then assessed. Once again, if imbalance remains, the propensity score model and/or matching algorithm must be re-specified. Following balance achievement, outcomes from time-dependent treatment can be compared across groups. In outcomes analysis, matched controls who later receive treatment are censored at their treatment date. If matching with replacement, not-yet-treated patients may be eligible for matching at multiple time points, including as treated patients at their treatment date [21]. Weighting is necessary when matching with replacement to avoid false imprecision.

To date, longitudinal matching methods have relied on iterative, manual re-specification to achieve covariate balance. Balance is not guaranteed and, consequently, nor is fulfillment of ignorability. To our knowledge, machine learning-based approaches have yet to be considered for automating balancing of time-dependent covariate histories when longitudinal matching.

#### Longitudinal genetic matching on time-dependent covariates

To reduce the need for manual adjustments when longitudinal matching, we propose a longitudinal extension of an established machine learning-based method, genetic algorithm matching on time-invariant covariates. Genetic algorithm matching, developed by Diamond & Sekhon in 2013, automates the process of optimizing time-invariant covariate balance through the use of an evolutionary genetic search algorithm [9, 22, 23].

Rather than measuring similarity across individuals based on differences in estimated propensity scores, genetic algorithm matching estimates a weighted form of a generalized Mahalanobis distance function:

5 $$Distance\left({x}_{i},{x}_{j},W\right)= \sqrt{{({x}_{i}-{x}_{j})}^{T}{(S}^{-1/2}{)}^{T}W{(S}^{-1/2}){(x}_{i}-{x}_{j})}$$

where *x*_*i*_ and *x*_*j*_ are time-invariant covariates measured at baseline for individuals *i* and *j*, respectively, *W* is a positive definite matrix of weights for which diagonal *K* parameters must be chosen and off diagonal parameters are 0, *S* is the sample covariance matrix of *X*, and *S*^*−1/2*^ is the Cholesky decomposition of *S* such that:

6 $$S={S}^{-1/2}({S}^{-1/2}{)}^{T}$$

A propensity score is recommended for inclusion alongside covariates when estimating the generalized Mahalanobis distance [9, 24]. To automate the process of iteratively checking covariate balance and respecifying matches, this approach employs an evolutionary genetic search algorithm to maximize covariate balance according to a user-specified loss function [9, 22]. The algorithm continuously proposes batches of weights that modify the distance metric, with each batch generated learning from the prior batch to improve performance. The algorithm continues to iterate until a minimum of the loss function is achieved. According to any loss function specified, genetic algorithm matching will asymptotically converge to the optimal matched cohort. The standard loss function evaluated by the algorithm maximizes *p*-values from bootstrapped Kolmogorov–Smirnov (KS) and paired t-tests for all variables, using lexical optimization for a fixed sample size specified within the optimization [25–27].

We extend this method in a sequential risk set matching framework. As in Lu (2005) [17], our longitudinal genetic matching extension begins by coarsening the study period into a series of time points, determined based on observed treatment dates and sample sizes for resulting risk sets. At each time *t*, patients in risk set *R*_*t *_are genetic algorithm matched based on the values of their fixed and time-dependent covariates using the generalized Mahalanobis distance metric (5). Within each risk set, the evolutionary genetic search algorithm will asymptotically converge to the optimal matched cohort for the user-specified loss function [22]. To provide reasonable starting values when initiating the evolutionary genetic search algorithm, we consider including a proxied time-dependent propensity score alongside covariates for estimating the generalized Mahalanobis distance at *t*.

In a cross-sectional setting, genetic algorithm matching outperformed traditional propensity score matching when balancing time-invariant covariates of interest and was consequently able to establish less biased effect estimates [10, 28]. In simulation, we explore whether extending this machine learning-based method to match patients over a longitudinal follow up period presents similar advantages.

## Methods

**Fig. 1:**
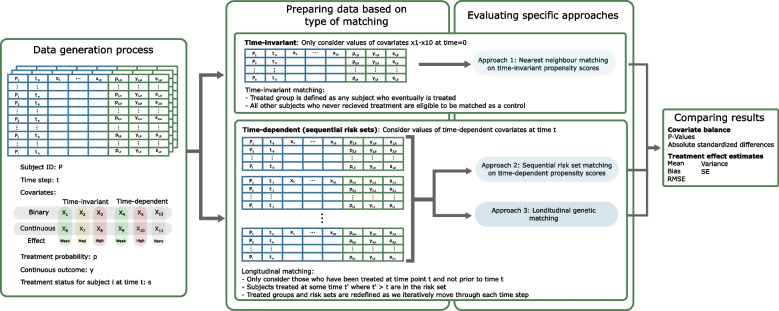
Overview of simulation framework and study design. Using Monte Carlo simulation, longitudinal datasets are generated with pre-specified covariate distributions and correlation structures before applying assumed treatment assignment and outcome models. Matching methods are then applied within each dataset using covariates observed either at baseline (time-invariant propensity score matching) or per time interval (time-dependent propensity score and genetic matching). Treatment effects are estimated in each matched cohort. The results of each matching method are then compared based on bias and efficiency of treatment effect estimates and covariate balance metrics

To compare the methods described above for mitigating time-dependent confounding and supporting real-world comparative evaluations, we undertook a Monte Carlo simulation study. Our simulation framework is depicted in Fig. 1. Simulated data reflected a static treatment assigned at multiple time points and considered a treatment model, a continuous outcome model, and covariates, varying in terms of their correlation with treatment status and/or the outcome, their time-dependency, and distributions. After simulation, we matched controls to treated patients using: (1) nearest neighbour matching on time-invariant, baseline propensity scores; (2) sequential risk set matching on proxied time-dependent propensity scores; and (3) longitudinal genetic matching on time-dependent covariates. We ascertained relative performance based on ability to balance time-invariant and time-dependent covariates and efficiently estimate unbiased treatment effects. Scenario analyses enabled an assessment of sensitivity to assumed data inputs and specifications. We conducted this simulation study using Python 3.7 and R version 3.6.2 [29, 30]. Matching was performed using the Matching package in R [31].

### Data generation

We based the design of our data generation process on three prior simulation studies: (1) Austin 2014 [32] comparing multiple algorithms for matching on time-invariant propensity scores; (2) Setoguchi, et al. 2008 [13] evaluating machine learning approaches for time-invariant propensity score estimation; and (3) Lu 2005 [17] assessing sequential and simultaneous matching on proxied time-dependent propensity scores.

Like Austin 2014 [32], we simulated a continuous outcome measure, *y*_*it*_, and twelve covariates, *x*_*k*_ for *k* = [1,… 12], affecting treatment selection and/or the outcome. Of these covariates, six were binary generated from a Bernoulli distribution with a parameter value of 0.5 and six were continuous covariates generated from a standard-normal distribution. Treatment effect, *θ*, was estimated using a linear regression and the true effect was assumed to be one:

$${y}_{it}={z}_{it}+{\alpha }_{L}{x}_{1i}+{\alpha }_{M}{x}_{2i}+{\alpha }_{H}{x}_{3i}+{\alpha }_{H}{x}_{5it}+{\alpha }_{L}{x}_{6i}+{\alpha }_{M}{x}_{7i}+{\alpha }_{H}{x}_{8i}+{\alpha }_{H}{x}_{10it}+{\alpha }_{L}{x}_{11it}+{\alpha }_{L}{x}_{12it}$$

where coefficients ${\alpha }_{L},{\alpha }_{M}, {\alpha }_{h}$ represented low, medium and high effects and were set to log (1.25), log(1.5) and log (1.75), respectively. Unlike Austin 2014 [32], our simulation study considered time-invariant covariates alongside time-dependent covariates.

Treatment status, *z*_*it*_, was assigned longitudinally over the study period and included time-dependent covariates affecting the probability of treatment assignment and/or the outcome. Following Lu 2005 [17], we assigned subject treatment status by sequentially simulating the true treatment probability, *p*_*i*_, based on a logistic regression model at each time, *t*:

$$Logit\left({p}_{it}\right)= {\alpha }_{0, treat}+{\alpha }_{L}{x}_{1i}+{\alpha }_{M}{x}_{2i}+{\alpha }_{H}{x}_{3i}+{\alpha }_{L}{x}_{4it}+{\alpha }_{H}{x}_{5it}+ {\alpha }_{M}({x}_{4it}{x}_{5it}) +{\alpha }_{L}{x}_{6i}+{\alpha }_{M}{x}_{7i}+{\alpha }_{L}{x}_{7i}^{2}+{\alpha }_{H}{x}_{8i}+{\alpha }_{L}{x}_{9it}^{2}+ {\alpha }_{M}({x}_{6i}{x}_{9it}^{2}) +{\alpha }_{H}{x}_{10it}$$

where ${\alpha }_{0, treat}$ was an intercept term adjusted to assign treatment to 1/3 of patients in our baseline scenario, as in Austin 2014 [32]. Treatment assignment was sampled from a Bernoulli distribution with parameter *p*_*it*_ and was restricted to at most once per subject. After treatment assignment in one interval, subjects were excluded from later time intervals to ensure that treatment assignment and risk set eligibility only depended on covariate histories and not future covariate values. Based on Setoguchi, et al. 2008 [13], our base case treatment assignment model assumed non-linearity and non-additivity.

Our simulation study period considered six time intervals, representing bi-monthly data collected over a one-year period. To allow for time dependencies, we set six covariates as time-dependent in our base case scenario. Of these, three time-dependent covariates were continuous and three were dichotomous with values that changed across intervals (on-offs). As in Lu 2005 [17], we considered first order autoregressive, AR(1), processes in our base case scenario to incorporate autocorrelation within continuous time-dependent covariate values and assumed independence across covariates. Through Monte Carlo simulation, we generated 1,000 datasets according to the above specifications, each consisting of 1,000 subjects.

### Statistical analysis

Within each simulated dataset, we applied each of the three matching methods described to select controls for treated patients. When considering the applicability of matching algorithms unable to explicitly consider time-dependencies, we assumed treatment status was constant and assigned at baseline and considered only baseline values for time-dependent covariates. In our base case scenario, we 1:1 matched patients on all time-dependent and time-invariant covariates simulated above, assuming an incorrect functional form for the estimated propensity score consistent with Setoguchi, et al. 2008 [13]. In all matching analyses, we allowed for replacement and ties.

For nearest neighbour matching on time-invariant propensity scores, we estimated each individual’s propensity score, $\widehat{\mathrm{Pr}\left({z}_{i}=1|{x}_{it0}\right)}$, based on the following incorrectly specified logistic regression model:

$$Logit\left({\widehat{p}}_{i}\right)= {\alpha }_{0, treat}+{\alpha }_{1}{x}_{1i}+{\alpha }_{2}{x}_{2i}+{\alpha }_{3}{x}_{3i}+{\alpha }_{4}{x}_{4it0}+{\alpha }_{5}{x}_{5it0}+{\alpha }_{6}{x}_{6i}+{\alpha }_{7}{x}_{7i}+{\alpha }_{8}{x}_{8i}+{\alpha }_{9}{x}_{9it0}+{\alpha }_{10}{x}_{10it0}$$

Sequential risk set matching on time-dependent propensity scores instead considered the following incorrectly specified Cox proportional hazards model to estimate proxied propensity scores:

$$h_i\left(t\right)=h_{0,i}\left(t\right)\exp\left(\alpha_{0,treat}+\alpha_1x_{1i}+\alpha_2x_{2i}+\alpha_3x_{3i}+\alpha_4x_{4it}+\alpha_5x_{5it}+\alpha_6x_{6i}+\alpha_7x_{7i}+\alpha_8x_{8i}+\alpha_9x_{9it}+\alpha_{10}x_{10it}\right)$$

Longitudinal genetic matching considered all time-dependent and time-invariant covariates as well as the proxied time-dependent propensity score estimated in (10) above. Optimization criteria for genetic matching involved maximizing p–values from bootstrapped KS tests and paired t-tests at time of matching, using lexical optimization for a fixed sample size specified within the optimization [25–27]. R code for our proposed longitudinal extension of genetic algorithm matching is provided in Supplemental Materials.

After identifying matched cohorts, we evaluated the following performance metrics at time of treatment across all datasets: (i) ability to balance measured covariates; (ii) mean treatment effect estimates: $\frac{1}{M}\sum_{m=1}^{M}{\theta }_{m}$, where M is the number of simulations; (iii) mean variance of treatment effect estimates: $\frac{1}{\mathrm{M}}\sum_{\mathrm{m}=1}^{\mathrm{M}}{\mathrm{S}}_{\mathrm{m}}^{2}$, where ${\mathrm{S}}_{\mathrm{m}}^{2}$ is the estimated variance within each simulation; (iv) mean standard error of treatment effect estimates: $\frac{1}{\mathrm{M}}\sum_{\mathrm{m}=1}^{\mathrm{M}}({\mathrm{S}}_{\mathrm{m}}/\sqrt{\mathrm{n}})$, where ${\mathrm{S}}_{\mathrm{m}}$ is the estimated standard deviation and n is the number of subjects within each simulation; (v) mean absolute bias of effect estimates: $\frac{1}{M}\sum_{m=1}^{M}{|(1.0-\theta }_{m})|$; (vi) mean bias of effect estimates: $\frac{1}{M}{\sum }_{m=1}^{M}(1.0-{\theta }_{m})$; and (vii) root mean squared error (RMSE) of effect estimates: $\frac{1}{M}\sum_{m=1}^{M}{{(1.0-\theta }_{m})}^{2}$.

Consistent with most longitudinal matching studies [5], we assessed balance of time-invariant and time-dependent covariates within each time interval through estimating means and boxplots of *p*-values from bootstrapped KS tests in continuous variables and paired t-tests in binary variables, as well as mean absolute standardized differences:

$$\frac{1}{M}\sum_{m=1}^{M}\left|({\overline{x} }_{{treated}_{m,t}}-{\overline{x} }_{{controls}_{m,t}})/\sqrt{{(s}_{{treated}_{m,t}}^{2}+{s}_{{controls}_{m,t}}^{2})/2}\right|$$

We also considered aggregate covariate balance, similar to Lu [17], through examining balance statistics at baseline and time of treatment, by pooling matched cohorts across time intervals. We re-estimated absolute standardized differences across all treated intervals and report means across simulated datasets. We also estimated paired t-tests and bootstrapped KS tests for binary and continuous covariates, respectively, and report corresponding means and boxplots.

In all analyses, we assumed a threshold of *p* < 0.05 for statistical significance and *p* < 0.10 for weak significance. Covariates with a *p*-value less than 0.05 were therefore strongly imbalanced, a *p*-value between 0.05 and 0.10 were weakly imbalanced, and above 0.10 were balanced. For absolute standardized differences, we applied common rules of thumb [33]. A value above 20 was strongly imbalanced, between 10 and 20 was weakly imbalanced, and below 10 was balanced. While balance of time-dependent covariates was primarily considered at time of treatment, we also estimated balance of these covariates (*x*_*4*_, *x*_*5*_, *x*_*9*_, and *x*_*10*_) at baseline (*t* = 0).

### Scenario analysis

To explore the sensitivity of our results to varying data inputs, specifications, and deviations from assumptions, we considered six additional scenarios (A to F), outlined in Table 1. In Scenario A, we assumed that propensity scores were estimated using correct functional forms, in contrast to our base case and all other scenarios where propensity score models failed to account for non-linearity and non-additivity in the true treatment assignment model. In Scenario B and C, we allow for pairwise correlation between all baseline and time-varying covariates ranging from weak, at 0.20, to strong, at 0.70. Scenario D explores a different autocorrelation structure for the time-dependent continuous covariates, assuming a first order moving average, MA(1), process. Scenarios E and F introduce covariates generated from non-standard normal distributions, including normal, gamma and Poisson distributions. Additional sensitivity analysis reported in Supplemental Materials explored the influence of sample size of generated datasets, alternative matching specifications, including propensity score definitions and optimization criteria, as well as outcome models on results (Supplemental Tables 4, 5, and 6, respectively).

**Table 1:** Summary of simulation scenarios

Scenario	Description	Covariate distributions	Treatment assignment model	Correlations
-	Base case	*x*_*1*_*, x*_*2*_*, x*_*3*_*, x*_*4*_*, x*_*5*_$$\sim Bernoulli (0.5)$$ *x*_*6*_*, x*_*7*_*, x*_*8*_*, x*_*9*_*, x*_*10*_$$\sim N(\mathrm{0,1})$$	$$Logit\left({p}_{it}\right)= {\alpha }_{0, treat}+{\alpha }_{L}{x}_{1i}+{\alpha }_{M}{x}_{2i}+{\alpha }_{H}{x}_{3i}+$$$${\alpha }_{L}{x}_{4it}+{\alpha }_{H}{x}_{5it}+ {\alpha }_{M}({x}_{4it}{x}_{5it}) +$$$${\alpha }_{L}{x}_{6i}+$$$${\alpha }_{M}{x}_{7i}+$$$${\alpha }_{L}{x}_{7i}^{2}+$$$${\alpha }_{H}{x}_{8i}+$$$${\alpha }_{L}{x}_{9it}^{2}+ {\alpha }_{M}({x}_{6i}{x}_{9it}^{2}) +$$$${\alpha }_{H}{x}_{10it}$$	Autocorrelation: *x*_*9*_*, x*_*10*:_ AR(1) Pairwise correlation: *ρ* = 0
A	Correct functional form	Same as base case	$$Logit\left({p}_{it}\right)= {\alpha }_{0, treat}+{\alpha }_{L}{x}_{1i}+{\alpha }_{M}{x}_{2i}+{\alpha }_{H}{x}_{3i}+$$$${\alpha }_{L}{x}_{4it}+{\alpha }_{H}{x}_{5it}+$$$${\alpha }_{L}{x}_{6i}+$$$${\alpha }_{M}{x}_{7i}+$$$${\alpha }_{H}{x}_{8i}+$$$${\alpha }_{L}{x}_{9it}+$$$${\alpha }_{H}{x}_{10it}$$	Same as base case
B	Weak pairwise correlation	Same as base case	Same as base case	*x*_*9*_*, x*_*10*:_ AR(1) All covariates: *ρ* = 0.2
C	Strong pairwise correlation	Same as base case	Same as base case	*x*_*9*_*, x*_*10*:_ AR(1) All covariates: *ρ* = 0.7
D	Different autocorrelation structure	Same as base case	Same as base case	*x*_*9*_*, x*_*10*:_ MA(1) *ρ* = 0
E	Non-standard normal covariate distributions	*x*_*1*_*, x*_*2*_*, x*_*3*_*, x*_*4*_*, x*_*5*_$\sim Bernoulli (0.5)$ *x*_*6*_*, x*_*7*_*, x*_*8*_$\sim N(\mathrm{0,1})$ *x*_*9*_*, x*_*10*_$\sim N(\mathrm{2,1})$	Same as base case	Same as base case
F	Non-normal covariate distributions	*x*_*1*_*, x*_*2*_*, x*_*3*_*, x*_*4*_*, x*_*5*_$\sim Bernoulli (0.5)$ *x*_*6*_*, x*_*7*_*, x*_*8*_$\sim N(\mathrm{0,1})$ *x*_*9*_$\sim Poisson(2)$ *x*_*10*_$\sim Gamma(\mathrm{2,1})$	Same as base case	Same as base case

Description of all simulation scenarios ranging from Scenario A to Scenario F, exploring sensitivity of results to different functional forms of the propensity score, pairwise correlation across covariates, autocorrelation within covariates, and covariate distributions

## Results

Our Monte Carlo simulation generated 1,000 simulated panel datasets for 1,000 individuals followed over six time intervals. In each interval, a mean of 60 patients were treated (95% CI: 27, 92), with an overall treatment rate of 1/3. Supplemental Fig. 1 depicts the proportions of patients who were treated over time, who were eligible for matching, and who were matched in the base case scenario using each of the three matching approaches: (1) nearest neighbour matching on time-invariant, baseline propensity scores; (2) sequential risk set matching on proxied time-dependent propensity scores; and (3) longitudinal genetic matching on time-dependent covariates.

### Covariate balance

**Fig. 2:**
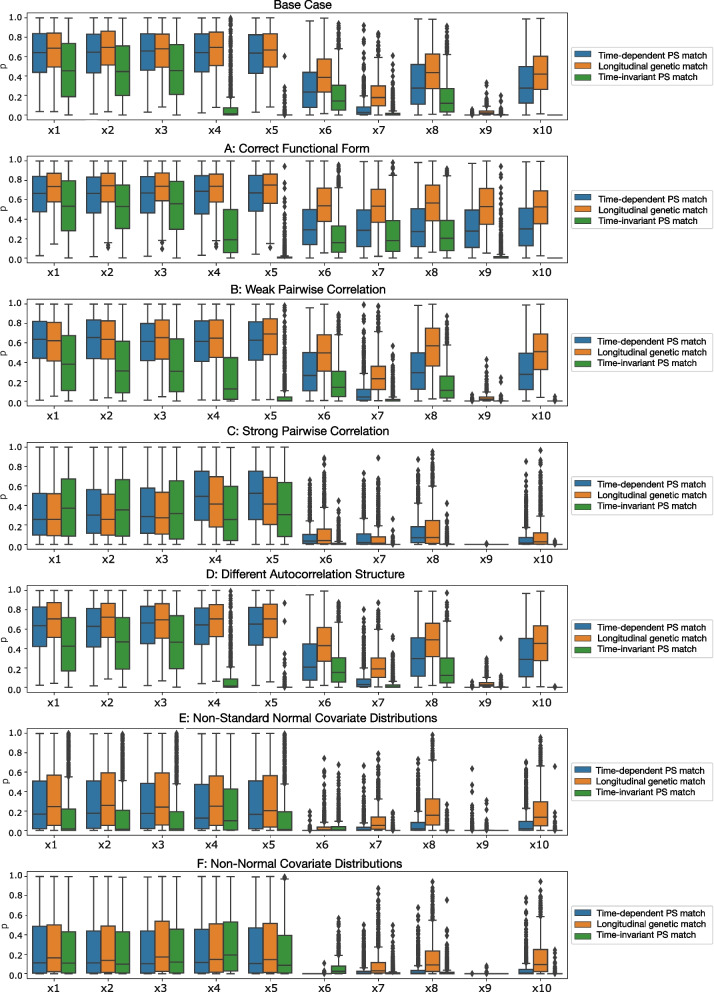
Per-covariate boxplots of *p*-values for each scenario

**Fig. 3:**
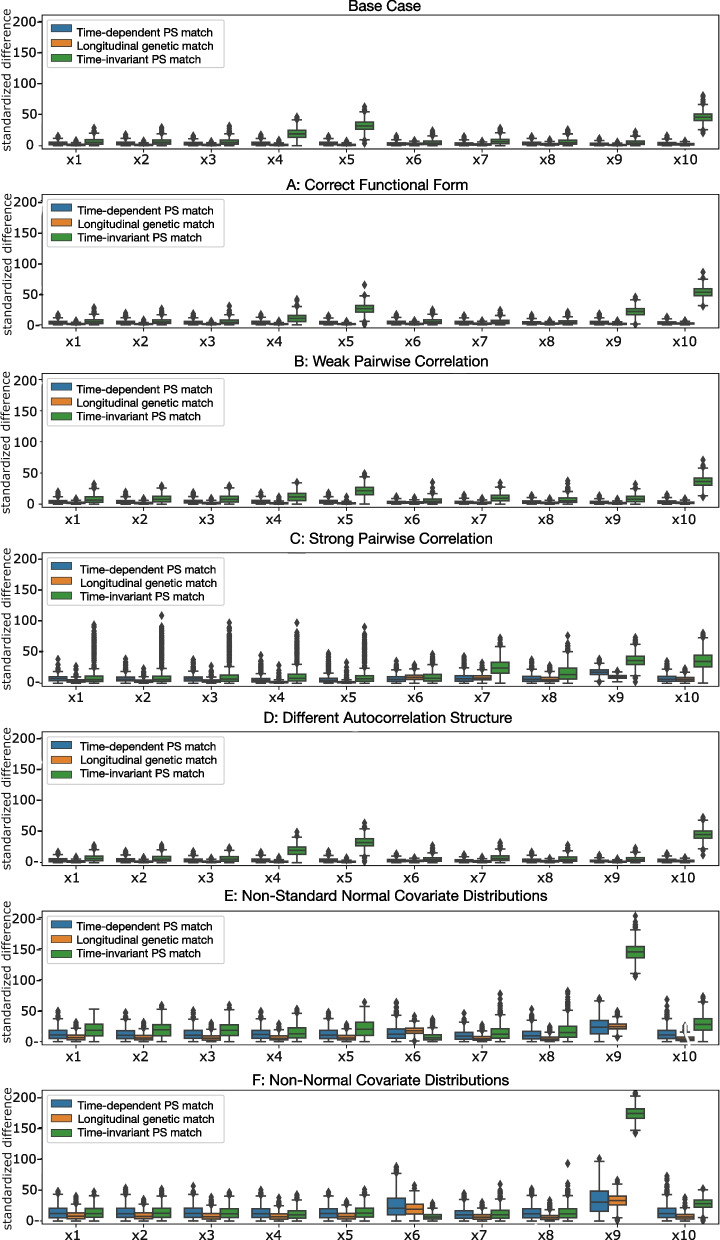
Per-covariate boxplots of mean absolute standardized differences for each scenario

Aggregate covariate balance achieved by each matching method at time of treatment varied across scenarios. Boxplots of *p*-values from distributional hypothesis tests as well as absolute standardized differences are reported in Figs. 2 and 3 respectively. Means and associated standard deviations are provided in Supplemental Tables 1 and 2. Across all scenarios and methods, imbalance occurred more frequently in continuous covariates and time-dependent covariates. Imbalance was most prevalent in the high pairwise correlation scenario (C) and the scenarios where covariate distributions were not standard normal (E and F). Considering mean *p*-values and standardized differences, the best balance metrics for all three matching methods occurred when the correct functional form of the propensity score model was estimated (scenario A), in which time-invariant matching balanced 6 of 10 covariates and both longitudinal methods balanced all 10 covariates at time of treatment.

Time-invariant propensity score matching performed worse than longitudinal matching in all scenarios, with the lowest balance observed in the high pairwise correlation scenario (C). In this scenario, all 10 covariates showed evidence of imbalance. Both longitudinal matching methods performed well in most scenarios, except for scenarios with non-standard normal covariate distributions (E and F). In these scenarios, longitudinal genetic matching outperformed time-dependent propensity score matching, on average achieving balance in 7 of 10 covariates compared to no covariates. In weak and strong pairwise correlation scenarios (B and C), longitudinal genetic matching balanced one more covariate than time-dependent propensity score matching.

The ability of each matching method to achieve covariate balance within each simulated time interval was consistent with aggregate findings. Both longitudinal matching methods improved covariate balance in each interval compared to before matching. Within-interval balance metrics for the base case scenario are depicted in Supplemental Figs. 2 and 3.

### Treatment effect estimation

**Fig. 4:**
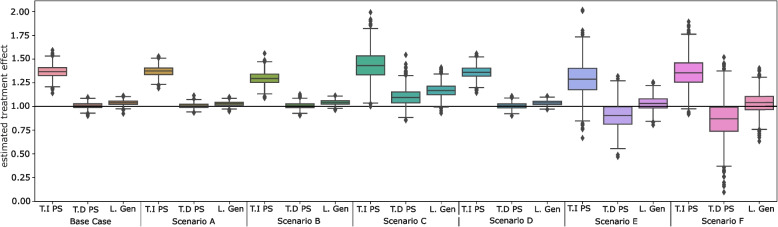
Boxplots of treatment effect estimates for each matching method across all scenarios

Figure 4 presents boxplots of treatment effect estimates for each of the three matching approaches, with means and corresponding variance reported in Supplemental Table 3. Across all methods and scenarios, the minimum observed treatment effect estimate was 0.91 and the maximum was 1.47 (9% downward and 47% upward bias respectively). The smallest and largest observed bias for each matching method was: 29% (scenario E) and 44% (scenario C) for time-invariant propensity score matching; 0.7% (scenario A) and 14% (scenario E) for time-dependent propensity score matching; and 3% (scenarios A and F) and 17% (scenario C) for longitudinal genetic matching. Notably, both time-dependent propensity score matching and longitudinal genetic matching performance improved when the correct functional form for the propensity score model was assumed (Scenario A). The accuracy of treatment effect estimates declined when strong pairwise correlation, or non-normal covariate distributions were introduced in the underlying data.

In all scenarios, longitudinal matching methods outperformed time-invariant propensity score matching, achieving lower bias, lower RMSE, and more efficient treatment effect estimates. Variance and standard errors of treatment effect estimates were lowest for longitudinal genetic matching in all scenarios indicating improved efficiency, except scenario A when the correct functional form of the proxied time-dependent propensity score was known. In the base case and in scenarios A to D, both longitudinal methods achieved similar bias and RMSE. Time-dependent propensity score matching showed slightly improved metrics compared to time-dependent genetic matching in the aforementioned scenarios (mean lower bias of 3.8% and mean lower RMSE of 0.02). Both longitudinal matching methods were robust to weak pairwise correlation (Scenario B). As pairwise correlation increased, both bias and RMSE increased, by 9.3% and 0.10 for time-dependent propensity score matching and by 13.3% and 0.14 for longitudinal genetic matching.

In scenarios E, and F, when covariate distributions deviated from standard normality, longitudinal genetic matching outperformed time-dependent propensity score matching resulting in 13% and 9% lower bias and 0.01 and 0.06 lower RMSE, respectively. Additional sensitivity analysis showed that longitudinal genetic matching performance improved with the inclusion of a propensity score and was sensitive to changes in the specified optimization criteria, with the standard loss function minimizing *p*-values from bootstrapped KS and paired t-tests performing best (Supplemental Table 5).

## Discussion

We propose a longitudinal extension of genetic algorithm matching for supporting real-world evaluations of time-dependent treatments. In simulation, we compare performance of longitudinal genetic matching with time-dependent propensity score matching and naïve matching on time-invariant, baseline propensity scores. Although the latter remains the most common approach for observational studies evaluating time-varying treatments [15], we find that baseline propensity score matching is unable to account for time-dependent confounding and results in biased treatment effect estimation in all scenarios. This finding aligns with past research comparing performance of baseline propensity score matching with alternative longitudinal matching and regression adjustment approaches [17, 34]. We also find that longitudinal genetic matching offers several advantages to time-dependent propensity score matching. In all tested scenarios, longitudinal genetic matching achieved comparable or better performance in terms of ability to balance covariates, bias and MSE of treatment effect estimates, without the need to iteratively, manually re-specify a time-dependent propensity score. In the presence of non-normally distributed covariates common for real-world health data [35], longitudinal genetic matching outperformed time-dependent propensity score matching. Genetic matching is a non-parametric approach that is less sensitive to distributional assumptions than propensity score matching, which in a time-invariant setting requires strong overlap of propensity score distributions to eliminate bias in the presence of non-normal covariates [36–38].

Our study responds to an unmet need for simulation frameworks that enable flexible comparisons of relative efficiency, bias and model sensitivity for competing longitudinal matching approaches. While existing simulation studies explore sensitivity of time-invariant matching methods to various data generation processes, matching specifications, and outcomes [32, 39–42], prior simulation studies of longitudinal matching methods are scarce. Those available conduct limited (if any) scenario analysis and assume simplistic data generation processes that may be unrealistic in real-world data, with a very small number of uncorrelated, normally distributed covariates, or linear, additive treatment assignment models [17, 21, 34]. Our framework builds on these published studies through explicitly modelling different aspects of a panel data generating mechanism and creating a series of pseudo experiments in which some patients are treated and other eligible at-risk patients are not, a design inherent to longitudinal matching. Our framework generates twelve binary and continuous covariates, that are either time-dependent or time-invariant and correlate with treatment status and/or the outcome. We consider a range of pairwise correlations across covariates, autocorrelation structures within time-dependent covariates, non-normal covariate distributions, and non-linearity and non-additivity in the propensity score model. Scenario analysis considers changes in assumed sample sizes, outcomes models, strengths of covariate associations, deviations from the correct functional form of the propensity score model, and modifications to longitudinal genetic matching specifications. Modifications will likely be necessary to ensure this framework is fit-for-purpose when evaluating alternate longitudinal matching approaches. Yet the data generation steps inherent to the framework are broadly generalizable as is the programming for testing sensitivity to underlying assumptions.

Our study newly develops a machine learning approach to longitudinal matching. While common in time-invariant propensity score literature [9, 13, 14, 43, 44], the advantages of machine learning-based approaches when automating balancing of time-dependent covariates are poorly established. In a cross-sectional setting, machine learning approaches, such as classification and regression trees (CART), random forests, neural networks and deep learning, vary in their ability to adjust for confounding and support stable, unbiased treatment effect estimation [14, 45, 46]. Future research is needed exploring whether alternative machine learning or deep learning approaches outperform genetic algorithm matching in a longitudinal setting.

While we applied extensive sensitivity analysis, including evaluating the influence of correlation, covariate distributions, outcome models, and longitudinal genetic matching specifications on results, certain modelling and data generation assumptions remain untested. Additional validation studies may inform modelling decisions and longitudinal matching specifications in the presence of heterogeneous outcome and covariate data, including simulation studies considering alternative types of outcomes and treatment assignment models and applied studies drawing on real-world data. While true treatment effects are rarely known in observational evaluations, applied studies will help establish the generalizability of simulation findings and may improve our understanding of real-world comparative performance. Our study is further limited by the computational intensity of simulating sequential genetic matching. Each modelled scenario of 1,000 simulations took approximately 3.75 days to run and future research examining performance of other machine learning algorithms in a longitudinal setting may require similar computational times. Yet within each dataset, the time required to longitudinally genetic match subjects was reasonable for practical use, with a run time of 5.25 min. We also tested performance sensitivity to sample sizes and found small decreases in treatment effect estimation for all three methods when sample size was reduced to 500 and small improvements when sample size increased to 2000, although conclusions about relative performance were unchanged (Supplemental Table 4). With a sample size of 500, bias increased by 0.8 percentage points for baseline propensity score matching, 0.7 percentage points for time-dependent propensity score matching, and 1.2 percentage points for longitudinal genetic matching. RMSE increased by 0.013 for baseline propensity score matching and 0.016 for both time-dependent propensity score matching and longitudinal genetic matching. Future research further exploring finite sample performance of longitudinal genetic matching with fewer observations or fewer time intervals would be beneficial. There are certain types of time-dependent treatments, which our framework was not developed to evaluate, such as treatment strategies that involve varying treatment status within patients over time. Modifications to the assumed treatment assignment model are required to extend our simulation framework to consider time-varying treatment status and evaluate associated methods, such as marginal structural models or modified sequential Cox models [47–49].

## Conclusions

When evaluating real-world impacts of static treatments whose assignment varies over time, longitudinal matching can mitigate time-dependent confounding and support causal inference. Traditional longitudinal matching relies on time-consuming, manual re-specifications to achieve covariate balance and meet ignorability. In contrast, machine learning using genetic matching automates balancing time-dependent covariate histories. Depending on underlying data generation processes, longitudinal genetic matching achieves similar or improved performance compared to time-dependent propensity score matching and is less sensitive to non-normal covariates. Our results demonstrate that longitudinal genetic matching can reliably inform future real-world assessments of time-dependent treatments.

### Supplementary Information

**Additional file 1: R code for our proposed longitudinal extension of genetic algorithm matching as well as the following supplemental figures and tables: Supplemental Figure 1.** Boxplots showing treatment rates and risk set eligibility over time in base case scenario.** Supplemental Table 1.** Aggregate covariate balance across scenarios represented as mean absolute standardized differences.** Supplemental Table 2.** Aggregate covariate balance across scenarios represented as *P*-values from bootstrapped KS tests and t-tests.** Supplemental Figure 2.** Per-interval mean absolute standardized differences, base case scenario.** Supplemental Figure 3.** Per-interval mean *P*-values from bootstrapped KS tests and t-tests, base case scenario.** Supplemental Table 3.** Bias and efficiency of treatment effects for each simulation scenario.** Supplemental Table 4.** Bias and efficiency of treatment effects with varying number of simulated subjects (500 and 2,000), base case scenario.** Supplemental Table 5.** Estimated treatment effects for additional scenarios evaluating the sensitivity of genetic matching to alternate specifications.** Supplemental Table 6.** Bias and efficiency of treatment effect estimates for alternative outcome models.

## Data Availability

Data supporting the findings of this study are available from the corresponding author upon request.
