# Supplementary material for: Genetic matching for time-dependent treatments: a longitudinal extension and simulation study
Source: BMC Med Res Methodol. 2023 Aug 9;23:181. doi: 10.1186/s12874-023-01995-5 (PMC10413721; doi:10.1186/s12874-023-01995-5)
Supplement: Supplementary file 1 — Additional file 1: R code for our proposed longitudinal extension of genetic algorithm matching as well as the following supplemental figures and tables: Supplemental Figure 1. Boxplots showing treatment rates and risk set eligibility over time in base case scenario. Supplemental Table 1. Aggregate covariate balance across scenarios represented as mean absolute standardized differences. Supplemental Table 2. Aggregate covariate balance across scenarios represented as P-values from bootstrapped KS tests and t-tests. Supplemental Figure 2. Per-interval mean absolute standardized differences, base case scenario. Supplemental Figure 3. Per-interval mean P-values from bootstrapped KS tests and t-tests, base case scenario. Supplemental Table 3. Bias and efficiency of treatment effects for each simulation scenario. Supplemental Table 4. Bias and efficiency of treatment effects with varying number of simulated subjects (500 and 2,000), base case scenario. Supplemental Table 5. Estimated treatment effects for additional scenarios evaluating the sensitivity of genetic matching to alternate specifications. Supplemental Table 6. Bias and efficiency of treatment effect estimates for alternative outcome models. [file 12874_2023_1995_MOESM1_ESM.docx]

**Supplemental Materials**

R code for our proposed longitudinal extension of genetic algorithm matching:

#---------------------------------------------------------------------------------------------------------------------------------------

# Implementation of longitudinal genetic matching through sequential risk-sets

#

# Extension of genetic matching done through the Matching package by J. Sekhon [1]: https://cran.r-project.org/web/packages/Matching/index.html

# Modifications to the parameters of the genetic algorithm can be made in accordance to the linked documentation above

#

# This example runs on a mock longitudinal dataset containing a variable representing the current time interval,

# 10 covariates (x1 to x10), a propensity score estimated through a cox proportional hazard model as in [2],

# a variable denoting treatment status at a given time interval, and an eligibility flag. Eligibility is defined

# as previously untreated or treatment as just been initiated at a given interval t.

#

# [1] Diamond A, Sekhon JS. Genetic matching for estimating causal effects: A general multivariate matching method for

# achieving balance in observational studies. Review of Economics and Statistics. 2013;95(3):932-945.

# [2] Lu B. Propensity score matching with time‐dependent covariates. Biometrics. 2005;61(3):721-728.

#

#

# Brandon Chan | June 2022

#---------------------------------------------------------------------------------------------------------------------------------------

# Set random seed

set.seed(2233)

# Load in packages

library(reticulate)

library(Matching)

# Load in mock dataset containing 10 covariates x1 to x10, a propensity score estimated by a

df <- read.csv("C:/Users/brchan/Documents/Github/time_varying_matching/v6/for_publication/mock_dataset_edit.csv")

# Define number of intervals in the dataset

nIntervals <- 6

# Initialize vectors to store indicies (row names) of treated and matched controls in addition to weights for the current iteration

treatedRowNamesGenMatch <- vector()

controlRowNamesGenMatch <- vector()

weightsGenMatch <- vector()

# Initialize lists to store output objects from gen match for each interval

genMatchIterationsLog <-list()

matchGenIterationsLog <- list()

matchBalanceGenIterationsLog <-list()

# Match in every time interval

for (t in 1:nIntervals){

# Identify eligible patients in interval t. Elibility defined as not previously treated or first treatment in interval t.

df_t = df[with(df, df$time == t & df$eligibility_flag == 1), ]

df_t_treated = df_t[df_t$treat_flag==1, ]

# Only run matching if there are treated and eligible subjects that exist in the interval

if (dim(df_t)[1] > 0 & dim(df_t_treated)[1] > 0){

tempGenMatch <- GenMatch(Tr=df_t$treat_flag,

X=df_t[c("x1", "x2", "x3", "x4", "x5", "x6", "x7", "x8", "x9", "x10", "pscore")],

pop.size=1000,

M=1,

verbose=FALSE,

print.level=0,

unif.seed=2233,

int.seed=2233)

tempGenMatchOut <- Match(Y=df_t$outcome,

Tr=df_t$treat_flag,

X=df_t[c("x1", "x2", "x3", "x4", "x5", "x6", "x7", "x8", "x9", "x10", "pscore")],

Weight.matrix=tempGenMatch,

ties=TRUE,

estimand="ATT")

tempGenMatchBalance <- MatchBalance(treat_flag ~ x1 + x2 + x3 + x4 + x5 + x6 + x7 + x8 + x9 + x10,

data=df_t,

match.out=tempGenMatchOut,

nboots=1000,

print.level=0)

# Log results

treatedRowNamesGenMatch <- append(treatedRowNamesGenMatch, as.double(row.names(df_t[tempGenMatchOut$index.treated, ])))

controlRowNamesGenMatch <- append(controlRowNamesGenMatch, as.double(row.names(df_t[tempGenMatchOut$index.control, ])))

weightsGenMatch <- append(weightsGenMatch, tempGenMatchOut$weights)

genMatchIterationsLog <- c(genMatchIterationsLog, setNames(list(tempGenMatch), t))

matchGenIterationsLog <- c(matchGenIterationsLog, setNames(list(tempGenMatchOut), t))

matchBalanceGenIterationsLog <- c(matchBalanceGenIterationsLog, setNames(list(tempGenMatchBalance), t))

} # End matching at interval t

} # End interval iteration loop (done all intervals)

# Use indices logged to segment dataframes in which matched controls and treated subjects are pairwise row by row.

df_treatedGen <- df[treatedRowNamesGenMatch,]

df_controlGen <- df[controlRowNamesGenMatch,]

Supplemental Figure 1. Boxplots showing treatment rates and risk set eligibility over time in base case scenario

Supplemental Table 1. Aggregate covariate balance across scenarios represented as mean absolute standardized differences

|  |  | Covariate, mean (SD) | | | | | | | | | | | | | |
| --- | --- | --- | --- | --- | --- | --- | --- | --- | --- | --- | --- | --- | --- | --- | --- |
| Scenario | Matching Method | X1 | X2 | X3 | X4 | X5 | X6 | X7 | X8 | X9 | X10 | X4 (trt) | X5 (trt) | X9 (trt) | X10 (trt) |
| Base case | Before Matching | 10.10 (6.18) | 16.97 (6.69) | 24.05 (7.05) | 5.92 (4.43) | 7.02 (4.98) | 68.22 (7.06) | 33.80 (6.37) | 48.66 (6.99) | 4.97 (3.88) | 7.52 (5.32) | - | - | - | - |
|  | Time-invariant PS matching | 6.82 (5.29) | 6.68 (5.00) | 6.50 (5.04) | 7.26 (5.44) | 7.01 (5.29) | 5.33 (4.15) | 7.34 (5.24) | 6.26 (4.84) | 5.89 (4.72) | 6.77 (5.47) | 19.06 (8.45) | 32.30 (8.65) | 5.32 (3.94) | 46.14 (8.23) |
|  | Time-dependent PS matching | 4.01 (3.00) | 4.01 (2.97) | 3.79 (2.91) | 6.01 (4.64) | 6.05 (4.54) | 3.26 (2.57) | 3.17 (2.38) | 3.92 (2.93) | 5.46 (4.09) | 6.02 (4.50) | 4.02 (3.09) | 3.85 (2.77) | 2.78 (2.09) | 3.59 (2.75) |
|  | Longitudinal genetic matching | 1.74 (1.40) | 1.66 (1.38) | 1.66 (1.31) | 5.56 (4.20) | 5.75 (4.42) | 3.20 (1.52) | 2.16 (1.42) | 2.51 (1.64) | 5.12 (3.83) | 5.46 (4.19) | 1.66 (1.40) | 1.55 (1.26) | 1.43 (1.09) | 2.62 (1.56) |
| A: Correct functional form | Before Matching | 11.61 (6.48) | 20.41 (6.84) | 28.73 (7.17) | 5.70 (4.24) | 6.58 (4.80) | 22.61 (6.50) | 41.48 (6.79) | 58.06 (7.05) | 5.94 (4.37) | 8.58 (5.65) | - | - | - | - |
|  | Time-invariant PS matching | 5.64 (4.37) | 5.57 (4.25) | 5.24 (4.25) | 5.79 (4.36) | 5.92 (4.38) | 5.72 (4.50) | 5.09 (3.87) | 4.47 (3.47) | 5.92 (4.53) | 5.95 (4.45) | 10.76 (6.86) | 25.84 (8.36) | 21.16 (7.58) | 52.56 (8.00) |
|  | Time-dependent PS matching | 3.86 (2.96) | 3.94 (3.08) | 3.90 (3.03) | 6.23 (4.73) | 6.05 (4.65) | 4.00 (2.95) | 3.82 (2.92) | 3.52 (2.58) | 6.06 (4.64) | 6.31 (4.67) | 3.84 (2.96) | 3.65 (2.80) | 3.81 (3.15) | 3.27 (2.49) |
|  | Longitudinal genetic matching | 1.47 (1.18) | 1.33 (1.11) | 1.35 (1.14) | 5.76 (4.58) | 5.72 (4.45) | 1.65 (1.30) | 1.92 (1.33) | 2.02 (1.23) | 5.81 (4.11) | 5.58 (4.23) | 1.36 (1.16) | 1.20 (1.03) | 1.72 (1.27) | 2.20 (1.23) |
| B: Weak pairwise correlation | Before Matching | 14.91 (6.72) | 9.20 (5.97) | 5.85 (4.43) | 19.93 (6.88) | 17.57 (6.71) | 81.77 (7.65) | 53.20 (7.00) | 66.50 (7.26) | 29.43 (6.62) | 35.86 (6.91) | - | - | - | - |
|  | Time-invariant PS matching | 8.11 (6.26) | 8.75 (6.19) | 8.58 (6.10) | 8.11 (6.22) | 8.12 (6.24) | 5.55 (4.37) | 10.17 (6.12) | 6.94 (5.42) | 7.69 (6.01) | 7.64 (6.13) | 12.14 (7.54) | 21.47 (8.75) | 8.88 (5.83) | 36.59 (8.72) |
|  | Time-dependent PS matching | 4.15 (3.13) | 4.02 (3.12) | 4.28 (3.12) | 6.30 (4.72) | 5.89 (4.50) | 3.22 (2.44) | 3.32 (2.49) | 3.58 (2.67) | 7.08 (5.15) | 5.47 (4.24) | 4.23 (3.09) | 4.14 (3.13) | 3.05 (2.24) | 3.65 (2.68) |
|  | Longitudinal genetic matching | 2.18 (1.67) | 2.02 (1.59) | 2.01 (1.63) | 5.56 (4.31) | 5.38 (3.94) | 2.78 (1.59) | 2.34 (1.56) | 2.22 (1.53) | 4.72 (3.65) | 5.01 (3.75) | 1.78 (1.54) | 1.53 (1.38) | 1.83 (1.37) | 2.34 (1.56) |
| C: Strong pairwise correlation | Before Matching | 94.37 (9.80) | 89.87 (9.61) | 86.07 (9.20) | 97.55 (9.94) | 95.39 (9.60) | 155.95 (9.90) | 125.16 (9.21) | 134.24 (8.70) | 115.90 (8.42) | 122.13 (8.60) | - | - | - | - |
|  | Time-invariant PS matching | 12.08 (17.65) | 12.95 (19.01) | 14.04 (19.19) | 11.17 (17.30) | 11.19 (17.59) | 10.04 (8.40) | 25.45 (12.88) | 16.70 (12.59) | 28.89 (12.84) | 18.28 (13.30) | 12.82 (15.69) | 11.22 (14.24) | 36.82 (10.32) | 35.58 (14.29) |
|  | Time-dependent PS matching | 7.39 (4.49) | 7.26 (5.00) | 7.41 (5.30) | 6.44 (6.96) | 7.59 (8.43) | 7.26 (5.44) | 8.47 (6.72) | 7.66 (6.23) | 24.52 (7.90) | 8.46 (6.62) | 5.78 (5.57) | 6.52 (7.40) | 17.85 (5.60) | 7.69 (5.92) |
|  | Longitudinal genetic matching | 3.74 (2.74) | 3.67 (2.74) | 3.72 (2.79) | 4.96 (4.23) | 5.21 (4.13) | 9.88 (5.23) | 9.06 (5.21) | 6.69 (5.04) | 12.50 (5.25) | 6.86 (5.30) | 2.04 (2.37) | 2.05 (2.55) | 10.29 (3.03) | 6.66 (4.24) |
| D: Different autocorrelation structure | Before Matching | 10.04 (6.16) | 17.16 (6.69) | 23.98 (6.84) | 6.18 (4.64) | 6.92 (5.02) | 68.15 (7.37) | 33.79 (6.60) | 47.86 (6.87) | 5.41 (4.12) | 9.65 (5.92) | - | - | - | - |
|  | Time-invariant PS matching | 7.14 (5.40) | 6.90 (5.22) | 6.61 (5.04) | 7.25 (5.36) | 7.60 (5.44) | 5.38 (4.26) | 7.55 (5.23) | 6.31 (4.82) | 6.01 (4.57) | 6.64 (5.40) | 19.34 (8.36) | 32.60 (8.81) | 5.39 (4.09) | 44.91 (8.96) |
|  | Time-dependent PS matching | 4.21 (3.12) | 4.35 (3.20) | 3.94 (3.01) | 6.19 (4.93) | 6.54 (5.03) | 3.47 (2.64) | 3.24 (2.40) | 3.78 (2.97) | 5.78 (4.47) | 5.95 (4.43) | 4.08 (2.99) | 3.96 (2.99) | 2.65 (1.96) | 3.68 (2.78) |
|  | Longitudinal genetic matching | 1.86 (1.56) | 1.74 (1.49) | 1.76 (1.40) | 5.77 (4.26) | 6.03 (4.47) | 2.99 (1.46) | 2.12 (1.47) | 2.32 (1.55) | 5.11 (3.88) | 5.55 (4.09) | 1.59 (1.36) | 1.43 (1.19) | 1.48 (1.16) | 2.57 (1.63) |
| E: Non-standard normal covariate distributions | Before Matching | 6.22 (4.51) | 8.81 (5.91) | 11.33 (6.08) | 5.41 (4.19) | 5.73 (4.37) | 197.04 (11.07) | 16.85 (6.43) | 22.20 (6.83) | 10.64 (5.94) | 5.92 (4.46) | - | - | - | - |
|  | Time-invariant PS matching | 19.19 (11.74) | 19.44 (12.04) | 19.07 (11.64) | 19.13 (11.55) | 19.32 (11.35) | 7.78 (6.50) | 14.58 (11.60) | 17.36 (13.73) | 19.85 (14.16) | 17.19 (14.51) | 14.87 (10.55) | 21.00 (13.18) | 144.84 (14.35) | 27.96 (13.02) |
|  | Time-dependent PS matching | 12.30 (8.90) | 12.10 (8.86) | 12.24 (8.75) | 13.35 (9.38) | 12.49 (9.15) | 14.55 (11.56) | 10.35 (7.69) | 11.49 (8.96) | 15.13 (10.20) | 12.43 (9.86) | 12.68 (8.86) | 12.21 (8.65) | 24.04 (14.41) | 12.61 (9.84) |
|  | Longitudinal genetic matching | 7.43 (5.64) | 6.95 (5.32) | 6.64 (5.23) | 7.99 (6.02) | 8.22 (6.16) | 17.48 (6.22) | 5.73 (4.58) | 5.15 (4.32) | 7.08 (5.38) | 7.75 (5.70) | 6.55 (5.02) | 7.00 (5.29) | 24.61 (6.10) | 5.05 (4.04) |
| F: Non-normal covariate distributions | Before Matching | 5.48 (4.18) | 7.23 (5.31) | 8.88 (5.58) | 5.25 (4.16) | 5.40 (4.05) | 196.80 (10.17) | 12.47 (6.11) | 15.93 (6.71) | 17.81 (6.13) | 6.65 (4.61) | - | - | - | - |
|  | Time-invariant PS matching | 13.61 (9.55) | 13.94 (9.83) | 13.47 (9.81) | 13.86 (9.92) | 13.51 (9.63) | 7.19 (5.19) | 12.19 (9.73) | 13.43 (10.65) | 27.88 (10.34) | 12.69 (9.97) | 11.31 (8.28) | 14.21 (9.71) | 172.47 (11.33) | 27.25 (9.25) |
|  | Time-dependent PS matching | 13.67 (10.07) | 14.11 (10.33) | 14.36 (10.42) | 14.30 (10.24) | 13.98 (10.26) | 24.44 (18.19) | 11.59 (9.12) | 13.76 (10.72) | 18.82 (12.36) | 14.22 (11.69) | 13.53 (9.87) | 13.39 (9.62) | 32.74 (21.51) | 14.68 (12.08) |
|  | Longitudinal genetic matching | 9.06 (7.16) | 8.89 (6.63) | 8.24 (6.51) | 8.54 (6.38) | 8.69 (6.36) | 19.41 (10.62) | 7.15 (5.39) | 6.47 (5.31) | 9.22 (6.61) | 8.06 (6.59) | 8.16 (6.50) | 8.50 (6.64) | 32.62 (10.16) | 7.43 (5.53) |

SD: standard deviation; PS: propensity score; trt: at time of treatment

Balance of time-dependent covariates (x4, x5, x9, and x10) described at baseline (t=1) and at time of treatment (pooled per-matched counterfactual).

Supplemental Table 2. Aggregate covariate balance across scenarios represented as P-values from bootstrapped KS tests and t-tests

|  |  | Covariates, mean (SD) | | | | | | | | | | | | | |  |
| --- | --- | --- | --- | --- | --- | --- | --- | --- | --- | --- | --- | --- | --- | --- | --- | --- |
| Scenario | Matching Method | X1 | X2 | X3 | X4 | X5 | X6 | X7 | X8 | X9 | X10 | X4 (trt) | X5 (trt) | X9 (trt) | X10 (trt) | |
| Base case | Before Matching | 0.26 (0.28) | 0.07 (0.15) | 0.01 (0.04) | 0.46 (0.30) | 0.40 (0.30) | 0.00 (0.00) | 0.00 (0.00) | 0.00 (0.00) | 0.44 (0.27) | 0.40 (0.30) |  |  |  |  | |
|  | Time-invariant PS matching | 0.46 (0.30) | 0.46 (0.30) | 0.47 (0.30) | 0.44 (0.30) | 0.45 (0.30) | 0.21 (0.20) | 0.02 (0.05) | 0.18 (0.19) | 0.13 (0.16) | 0.17 (0.18) | 0.09 (0.19) | 0.00 (0.03) | 0.00 (0.01) | 0.00 (0.00) | |
|  | Time-dependent PS matching | 0.62 (0.24) | 0.62 (0.24) | 0.64 (0.24) | 0.50 (0.29) | 0.49 (0.29) | 0.29 (0.24) | 0.07 (0.11) | 0.33 (0.25) | 0.16 (0.19) | 0.27 (0.25) | 0.62 (0.25) | 0.62 (0.24) | 0.00 (0.00) | 0.33 (0.25) | |
|  | Longitudinal genetic matching | 0.66 (0.23) | 0.67 (0.23) | 0.66 (0.22) | 0.49 (0.29) | 0.48 (0.29) | 0.41 (0.22) | 0.21 (0.15) | 0.45 (0.23) | 0.45 (0.27) | 0.49 (0.28) | 0.67 (0.23) | 0.66 (0.23) | 0.03 (0.03) | 0.44 (0.22) | |
| A: Correct functional form | Before Matching | 0.21 (0.26) | 0.03 (0.09) | 0.00 (0.02) | 0.48 (0.29) | 0.43 (0.30) | 0.04 (0.10) | 0.00 (0.00) | 0.00 (0.00) | 0.47 (0.30) | 0.35 (0.29) |  |  |  |  | |
|  | Time-invariant PS matching | 0.53 (0.29) | 0.53 (0.28) | 0.54 (0.28) | 0.52 (0.28) | 0.51 (0.29) | 0.22 (0.21) | 0.24 (0.21) | 0.25 (0.21) | 0.23 (0.21) | 0.20 (0.19) | 0.30 (0.29) | 0.03 (0.08) | 0.03 (0.09) | 0.00 (0.00) | |
|  | Time-dependent PS matching | 0.64 (0.24) | 0.64 (0.24) | 0.64 (0.24) | 0.50 (0.29) | 0.51 (0.30) | 0.34 (0.25) | 0.33 (0.24) | 0.33 (0.25) | 0.27 (0.25) | 0.25 (0.23) | 0.65 (0.24) | 0.65 (0.23) | 0.32 (0.24) | 0.34 (0.25) | |
|  | Longitudinal genetic matching | 0.72 (0.19) | 0.72 (0.20) | 0.72 (0.20) | 0.50 (0.30) | 0.50 (0.30) | 0.55 (0.22) | 0.54 (0.22) | 0.56 (0.22) | 0.50 (0.28) | 0.52 (0.28) | 0.71 (0.20) | 0.72 (0.20) | 0.53 (0.23) | 0.53 (0.22) | |
| B: Weak pairwise correlation | Before Matching | 0.11 (0.19) | 0.30 (0.29) | 0.47 (0.30) | 0.04 (0.10) | 0.06 (0.13) | 0.00 (0.00) | 0.00 (0.00) | 0.00 (0.00) | 0.00 (0.02) | 0.00 (0.00) |  |  |  |  | |
|  | Time-invariant PS matching | 0.41 (0.31) | 0.37 (0.31) | 0.38 (0.31) | 0.40 (0.31) | 0.40 (0.31) | 0.20 (0.19) | 0.02 (0.06) | 0.17 (0.17) | 0.10 (0.14) | 0.14 (0.16) | 0.25 (0.29) | 0.06 (0.14) | 0.00 (0.01) | 0.00 (0.00) | |
|  | Time-dependent PS matching | 0.61 (0.25) | 0.62 (0.25) | 0.60 (0.24) | 0.48 (0.29) | 0.50 (0.28) | 0.32 (0.24) | 0.09 (0.13) | 0.33 (0.25) | 0.14 (0.17) | 0.27 (0.24) | 0.60 (0.25) | 0.61 (0.25) | 0.00 (0.00) | 0.32 (0.24) | |
|  | Longitudinal genetic matching | 0.61 (0.25) | 0.62 (0.24) | 0.63 (0.25) | 0.48 (0.30) | 0.48 (0.29) | 0.50 (0.24) | 0.26 (0.18) | 0.56 (0.24) | 0.45 (0.27) | 0.51 (0.27) | 0.63 (0.25) | 0.66 (0.24) | 0.03 (0.04) | 0.51 (0.23) | |
| C: Strong pairwise correlation | Before Matching | 0.00 (0.00) | 0.00 (0.00) | 0.00 (0.00) | 0.00 (0.00) | 0.00 (0.00) | 0.00 (0.00) | 0.00 (0.00) | 0.00 (0.00) | 0.00 (0.00) | 0.00 (0.00) |  |  |  |  | |
|  | Time-invariant PS matching | 0.40 (0.32) | 0.39 (0.32) | 0.37 (0.32) | 0.42 (0.32) | 0.42 (0.31) | 0.02 (0.04) | 0.00 (0.01) | 0.01 (0.03) | 0.00 (0.00) | 0.01 (0.03) | 0.34 (0.31) | 0.37 (0.31) | 0.00 (0.00) | 0.00 (0.00) | |
|  | Time-dependent PS matching | 0.33 (0.28) | 0.36 (0.29) | 0.36 (0.29) | 0.48 (0.30) | 0.45 (0.31) | 0.08 (0.10) | 0.08 (0.13) | 0.12 (0.14) | 0.00 (0.01) | 0.12 (0.14) | 0.50 (0.29) | 0.50 (0.30) | 0.00 (0.00) | 0.06 (0.10) | |
|  | Longitudinal genetic matching | 0.33 (0.28) | 0.33 (0.28) | 0.34 (0.28) | 0.48 (0.30) | 0.45 (0.30) | 0.11 (0.16) | 0.07 (0.12) | 0.16 (0.20) | 0.01 (0.04) | 0.16 (0.19) | 0.46 (0.30) | 0.46 (0.30) | 0.00 (0.00) | 0.10 (0.16) | |
| D: Different autocorrelation structure | Before Matching | 0.27 (0.28) | 0.07 (0.14) | 0.01 (0.06) | 0.45 (0.30) | 0.41 (0.30) | 0.00 (0.00) | 0.00 (0.00) | 0.00 (0.00) | 0.42 (0.28) | 0.31 (0.29) |  |  |  |  | |
|  | Time-invariant PS matching | 0.45 (0.30) | 0.46 (0.30) | 0.47 (0.30) | 0.44 (0.30) | 0.42 (0.30) | 0.21 (0.20) | 0.03 (0.06) | 0.19 (0.19) | 0.15 (0.18) | 0.18 (0.19) | 0.09 (0.17) | 0.01 (0.04) | 0.00 (0.02) | 0.00 (0.00) | |
|  | Time-dependent PS matching | 0.62 (0.25) | 0.60 (0.25) | 0.63 (0.24) | 0.50 (0.29) | 0.48 (0.30) | 0.28 (0.24) | 0.07 (0.11) | 0.34 (0.25) | 0.16 (0.18) | 0.26 (0.24) | 0.62 (0.24) | 0.62 (0.24) | 0.00 (0.00) | 0.33 (0.25) | |
|  | Longitudinal genetic matching | 0.68 (0.23) | 0.68 (0.23) | 0.68 (0.22) | 0.49 (0.29) | 0.47 (0.29) | 0.45 (0.23) | 0.22 (0.15) | 0.50 (0.23) | 0.47 (0.28) | 0.51 (0.28) | 0.67 (0.23) | 0.68 (0.23) | 0.04 (0.04) | 0.47 (0.23) | |
| E: Non-standard normal covariate distributions | Before Matching | 0.45 (0.30) | 0.32 (0.30) | 0.21 (0.26) | 0.50 (0.29) | 0.48 (0.29) | 0.00 (0.00) | 0.07 (0.13) | 0.04 (0.10) | 0.24 (0.26) | 0.46 (0.29) |  |  |  |  | |
|  | Time-invariant PS matching | 0.16 (0.26) | 0.16 (0.26) | 0.16 (0.26) | 0.16 (0.26) | 0.15 (0.26) | 0.04 (0.08) | 0.00 (0.02) | 0.01 (0.02) | 0.00 (0.02) | 0.01 (0.03) | 0.24 (0.29) | 0.15 (0.25) | 0.00 (0.00) | 0.00 (0.02) | |
|  | Time-dependent PS matching | 0.29 (0.30) | 0.29 (0.31) | 0.28 (0.30) | 0.26 (0.29) | 0.28 (0.31) | 0.00 (0.01) | 0.03 (0.07) | 0.07 (0.11) | 0.04 (0.10) | 0.07 (0.12) | 0.27 (0.30) | 0.28 (0.30) | 0.01 (0.03) | 0.07 (0.12) | |
|  | Longitudinal genetic matching | 0.33 (0.30) | 0.34 (0.31) | 0.34 (0.31) | 0.38 (0.30) | 0.38 (0.31) | 0.03 (0.07) | 0.10 (0.13) | 0.22 (0.21) | 0.21 (0.20) | 0.24 (0.23) | 0.33 (0.31) | 0.31 (0.30) | 0.00 (0.02) | 0.20 (0.19) | |
| F: Non-normal covariate distributions | Before Matching | 0.49 (0.29) | 0.40 (0.31) | 0.31 (0.29) | 0.51 (0.30) | 0.49 (0.29) | 0.00 (0.00) | 0.17 (0.22) | 0.12 (0.19) | 0.07 (0.13) | 0.43 (0.29) |  |  |  |  | |
|  | Time-invariant PS matching | 0.25 (0.30) | 0.24 (0.29) | 0.26 (0.30) | 0.25 (0.30) | 0.25 (0.30) | 0.06 (0.09) | 0.02 (0.04) | 0.02 (0.06) | 0.01 (0.04) | 0.02 (0.06) | 0.30 (0.30) | 0.23 (0.28) | 0.00 (0.00) | 0.00 (0.01) | |
|  | Time-dependent PS matching | 0.26 (0.31) | 0.25 (0.29) | 0.24 (0.29) | 0.24 (0.30) | 0.25 (0.30) | 0.00 (0.00) | 0.03 (0.07) | 0.04 (0.08) | 0.02 (0.06) | 0.04 (0.09) | 0.26 (0.30) | 0.26 (0.31) | 0.00 (0.00) | 0.04 (0.09) | |
|  | Longitudinal genetic matching | 0.30 (0.32) | 0.27 (0.30) | 0.29 (0.31) | 0.35 (0.31) | 0.34 (0.30) | 0.00 (0.00) | 0.09 (0.13) | 0.16 (0.18) | 0.14 (0.20) | 0.18 (0.20) | 0.28 (0.31) | 0.28 (0.30) | 0.00 (0.00) | 0.17 (0.19) | |

SD: standard deviation; PS: propensity score; trt: at time of treatment

Balance of time-dependent covariates (x4, x5, x9, and x10) are described at both baseline (t=1) and at time of treatment (pooled respectively per-matched counterfactual). P-values estimated from bootstrapped Kolmogorov-Smirnov (KS) tests for continuous covariates and paired t-tests for binary covariates for a fixed sample size specified within the optimization.

Supplemental Figure 2: Per-interval mean absolute standardized differences, base case scenario


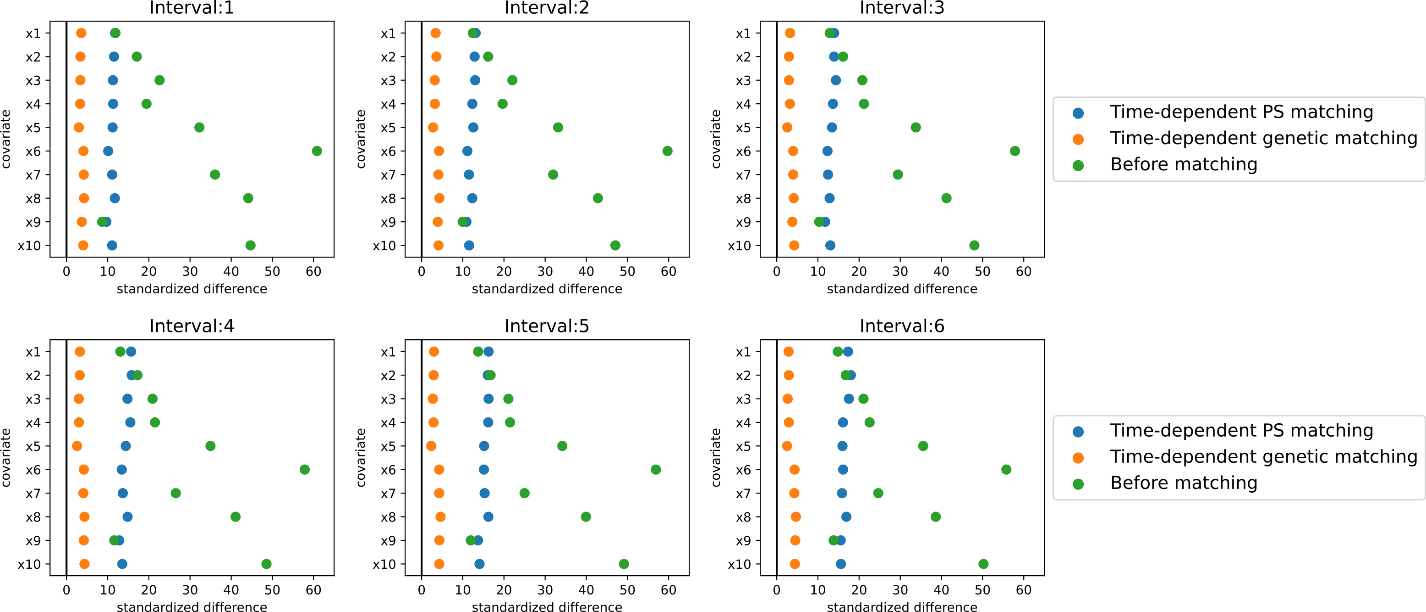


Mean per-interval absolute standardized differences across all 1000 simulated datasets for the base case scenario. Covariate balance in each interval estimated prior to matching and after longitudinal matching methods. Balance of time-dependent covariates (x4, x5, x9, and x10) are taken at time of treatment. Covariate balance improved after matching using either method.

Supplemental Figure 3: Per-interval mean P-values from bootstrapped KS tests and t-tests, base case scenario


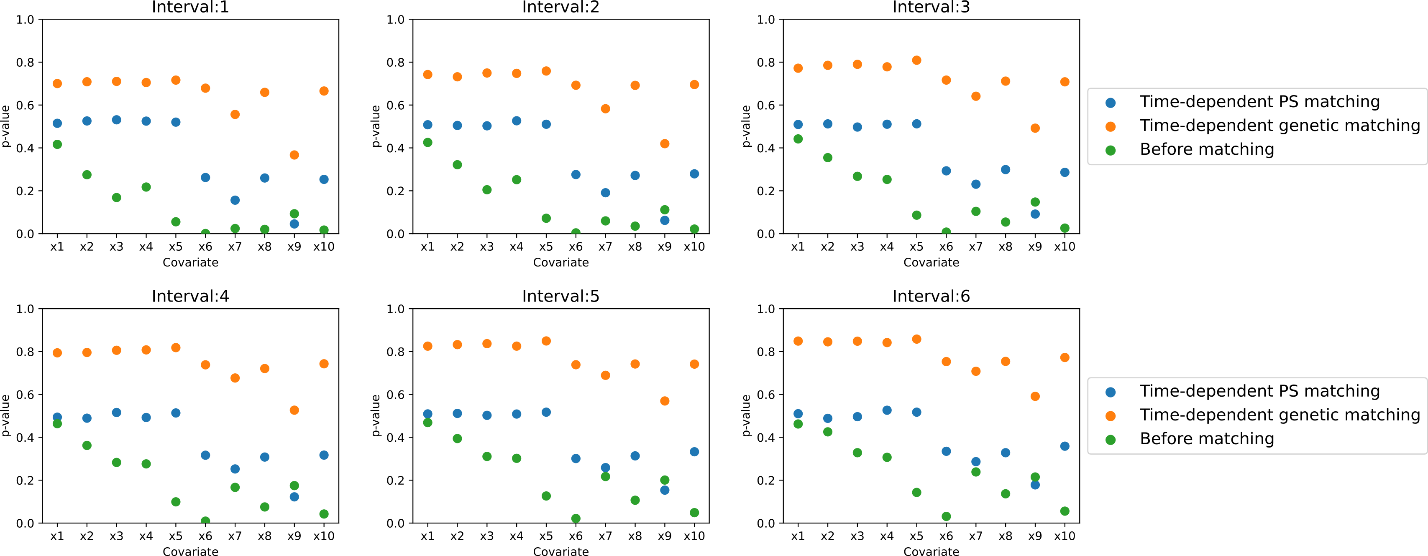


Mean per-interval P-values across all 1000 simulated datasets for the base case scenario. P-values estimated from bootstrapped KS tests for continuous covariates and paired t-tests for binary covariates for a fixed sample size specified within the optimization. Covariate balance in each interval estimated prior to matching and after longitudinal matching methods. Balance of time-dependent covariates (x4, x5, x9, and x10) are taken at time of treatment. Covariate balance improved after matching using either method.

Supplemental Table 3. Bias and efficiency of treatment effects for each simulation scenario

| Scenario | Matching Method | Mean | Variance | SE | RMSE | Abs.Bias | Bias |
| --- | --- | --- | --- | --- | --- | --- | --- |
| Base case | Time-invariant PS matching | 1.368 | 0.003 | 0.002 | 0.373 | 0.368 | -0.368 |
|  | Time-dependent PS matching | 1.008 | 0.002 | 0.001 | 0.032 | 0.025 | -0.008 |
|  | Longitudinal genetic matching | 1.040 | 0.001 | 0.001 | 0.046 | 0.041 | -0.040 |
| A: Correct functional form | Time-invariant PS matching | 1.372 | 0.003 | 0.002 | 0.375 | 0.372 | -0.372 |
|  | Time-dependent PS matching | 1.007 | 0.001 | 0.001 | 0.026 | 0.021 | -0.007 |
|  | Longitudinal genetic matching | 1.030 | 0.001 | 0.001 | 0.037 | 0.032 | -0.030 |
| B: Weak pairwise correlation | Time-invariant PS matching | 1.297 | 0.003 | 0.002 | 0.304 | 0.297 | -0.297 |
|  | Time-dependent PS matching | 1.009 | 0.002 | 0.001 | 0.032 | 0.025 | -0.009 |
|  | Longitudinal genetic matching | 1.042 | 0.001 | 0.001 | 0.049 | 0.043 | -0.042 |
| C: Strong pairwise correlation | Time-invariant PS matching | 1.439 | 0.003 | 0.002 | 0.464 | 0.439 | -0.439 |
|  | Time-dependent PS matching | 1.101 | 0.002 | 0.002 | 0.136 | 0.110 | -0.101 |
|  | Longitudinal genetic matching | 1.173 | 0.002 | 0.001 | 0.186 | 0.173 | -0.173 |
| D: Different autocorrelation structure | Time-invariant PS matching | 1.361 | 0.004 | 0.002 | 0.367 | 0.361 | -0.361 |
|  | Time-dependent PS matching | 1.008 | 0.002 | 0.001 | 0.033 | 0.026 | -0.008 |
|  | Longitudinal genetic matching | 1.038 | 0.001 | 0.001 | 0.045 | 0.039 | -0.038 |
| E: Non-standard normal covariate distributions | Time-invariant PS matching | 1.289 | 0.006 | 0.002 | 0.339 | 0.297 | -0.289 |
|  | Time-dependent PS matching | 0.907 | 0.006 | 0.002 | 0.164 | 0.133 | 0.093 |
|  | Longitudinal genetic matching | 1.032 | 0.003 | 0.002 | 0.079 | 0.063 | -0.032 |
| F: Non-normal covariate distributions | Time-invariant PS matching | 1.360 | 0.008 | 0.003 | 0.393 | 0.361 | -0.360 |
|  | Time-dependent PS matching | 0.863 | 0.009 | 0.003 | 0.247 | 0.197 | 0.137 |
|  | Longitudinal genetic matching | 1.036 | 0.004 | 0.002 | 0.119 | 0.093 | -0.036 |

RMSE: root mean squared error; PS: propensity score; SE: standard error; Abs.: absolute

In the base case and scenarios A through D, time-dependent propensity score matching produced the lowest bias and RMSE for mean treatment effects compared to the other methods. Longitudinal genetic matching produced the lowest variability in estimates across all scenarios in addition to having the lowest bias and RMSE in scenarios D and E, were non-standard normal covariate distributions were considered. Time-invariant propensity score matching produced the highest bias and RMSE across all scenarios.

Supplemental Table 4. Bias and efficiency of treatment effects with varying number of simulated subjects (500 and 2,000), base case scenario

| Scenario | Matching Method | Mean | Variance | SE | RMSE | Abs.Bias | Bias |
| --- | --- | --- | --- | --- | --- | --- | --- |
| Base case with n=1000 subjects | Time-invariant PS matching | 1.368 | 0.003 | 0.002 | 0.373 | 0.368 | -0.368 |
|  | Time-dependent PS matching | 1.008 | 0.002 | 0.001 | 0.032 | 0.025 | -0.008 |
|  | Longitudinal genetic matching | 1.040 | 0.001 | 0.001 | 0.046 | 0.041 | -0.040 |
| Base case with n=500 subjects | Time-invariant PS matching | 1.376 | 0.007 | 0.003 | 0.386 | 0.376 | -0.376 |
|  | Time-dependent PS matching | 1.015 | 0.003 | 0.002 | 0.048 | 0.038 | -0.015 |
|  | Longitudinal genetic matching | 1.052 | 0.003 | 0.002 | 0.062 | 0.053 | -0.052 |
| Base case with n=2000 subjects | Time-invariant PS matching | 1.364 | 0.002 | 0.001 | 0.366 | 0.364 | -0.364 |
|  | Time-dependent PS matching | 1.005 | 0.001 | 0.001 | 0.021 | 0.016 | -0.005 |
|  | Longitudinal genetic matching | 1.029 | 0.0004 | 0.001 | 0.034 | 0.030 | -0.030 |

RMSE: root mean squared error; PS: propensity score; SE: standard error; Abs.: absolute

To assess sensitivity to small sample sizes, we varied the number of simulated subjects to 500 or 2,000 compared to the primary scenario of 1,000. Results showed that with a lower population size, bias of estimates increased by 0.8 percentage points, 0.7 percentage points, and 1.2 percentage points for time-invariant propensity score matching, time-dependent propensity score matching, and longitudinal genetic matching respectively. These magnitudes correspond to a 2%, 92%, and 31% increase in bias. Variance increased by 105%, 116%, and 148% with a lower population size for time-invariant propensity score matching, time-dependent propensity score matching, and longitudinal genetic matching respectively. In contrast, a larger population size reduced bias by 0.4 percentage points, 0.3 percentage points, and 1.0 percentage points for time-invariant propensity score matching, time-dependent propensity score matching, and longitudinal genetic matching, respectively. Changed correspond to a 1%, 35%, and 25% reduction in bias, respectively. Efficiency also improved with a larger sample size, with a 51%, 52%, and 61% reduction in variance for time-invariant propensity score matching, time-dependent propensity score matching, and longitudinal genetic matching. While magnitudes of bias and variance changed depending on sample sizes, relative performance across longitudinal matching methods and time-invariant propensity score matching held, with no method appearing especially sensitive to this change.

Supplemental Table 5. Estimated treatment effects for additional scenarios evaluating the sensitivity of genetic matching to alternate specifications

| Scenario | Number of datasets | Matching Method | Mean | Variance | SE | RMSE | Abs.Bias | Bias |
| --- | --- | --- | --- | --- | --- | --- | --- | --- |
| Base case | n=1000 | Longitudinal genetic matching | 1.008 | 0.002 | 0.002 | 0.032 | 0.041 | -0.040 |
| Not including a propensity score | n=500 | Longitudinal genetic matching | 1.138 | 0.002 | 0.001 | 0.142 | 0.138 | -0.138 |
| Alternate propensity score (log proportional hazard rather than linear predictor, XB) | n=500 | Longitudinal genetic matching | 1.036 | 0.001 | 0.001 | 0.043 | 0.038 | -0.036 |
| Changing optimization criterion to focus on standardized differences | n=500 | Longitudinal genetic matching | 1.084 | 0.001 | 0.001 | 0.088 | 0.084 | -0.084 |

RMSE: root mean squared error; SE: standard error; Abs.: absolute

We explored the sensitivity of our proposed longitudinal matching method, sequential risk set genetic matching, to alternate specifications assuming our base case scenario covariate distributions, treatment assignment model, and outcome model. This exploratory analysis simulated 500 datasets, rather than 1,000, to reduce run-times. In our base case analysis, a Cox regression-based propensity score was included in based on past literature showing that in a time-invariant setting, inclusion of a propensity score improves genetic matching performance through provision of reasonable starting values.^1^ We tested sensitivity to this assumption through exclusion of a propensity score and found worse performance, through increased bias and RMSE.

There is some variation in the literature describing time-dependent propensity score estimation using Cox proportional hazards regression. While Lu 2005^2^ specifies that the propensity score is the estimated hazard function at time *t,* applied researchers tend to use the linear predictor when estimating the propensity score.^3,4^ For a distance metric that reflects the difference between two propensity scores, these estimates give equivalent distances:

$${Distance\left( x_{it},x_{jt} \right)= h}_{i}\left( t \right)- h_{j}\left( t \right)$$

$=(h_{0}\left( t \right)\exp\left( {\beta_{k}}^{'}x_{i}\left( t \right) \right)-h_{0}\left( t \right)\exp\left( {\beta_{k}}^{'}x_{j}\left( t \right) \right))$

$={\beta_{k}}^{'}x_{i}(t)-$ ${\beta_{k}}^{'}x_{j}(t)$

For a generalized Mahalanobis distance metric, this is not necessarily true. In scenario analysis, we considered sensitivity to a different propensity score function (the log proportional hazard) rather than the linear predictor. We found small increases in bias and RMSE when this alternate representation of the propensity score was included for longitudinal genetic matching.

Finally, when genetic matching, the user pre-specifies optimization criteria for the evolutionary genetic search algorithm. Using the genetic matching function in the Matching package for R,^5^ the default criterion maximizes p–values from bootstrapped KS tests and paired t-tests for a fixed sample size specified within the optimization. This default optimization criterion was used in our base case scenario, but we also tested sensitivity to alternate criteria, particularly minimizing standardized differences. Specifically, we considered calculating mean standardized differences based on the empirical quantile-quantile plot for each variable and minimizing this difference across variables using lexical optimization. This change adversely affected performance, resulting in small increases in bias and RMSE.

Supplemental Table 6. Bias and efficiency of treatment effect estimates for alternative outcome models

| Outcome Model | Matching Method | Mean | Variance | SE | RMSE | Abs.Bias | Bias |
| --- | --- | --- | --- | --- | --- | --- | --- |
| Base case outcome model | Time-invariant PS matching | 1.368 | 0.003 | 0.002 | 0.373 | 0.368 | -0.368 |
|  | Time-dependent PS matching | 1.008 | 0.002 | 0.001 | 0.032 | 0.025 | -0.008 |
|  | Longitudinal genetic matching | 1.040 | 0.001 | 0.001 | 0.046 | 0.041 | -0.040 |
| 1: Testing sensitivity of outcome to the presence of random error | Time-invariant PS matching | 1.367 | 0.004 | 0.002 | 0.373 | 0.367 | -0.367 |
|  | Time-dependent PS matching | 1.008 | 0.002 | 0.001 | 0.036 | 0.029 | -0.008 |
|  | Longitudinal genetic matching | 1.039 | 0.001 | 0.001 | 0.050 | 0.042 | -0.039 |
| 2: Testing sensitivity to inclusion of covariates that factor into treatment assignment model | Time-invariant PS matching | 1.388 | 0.005 | 0.002 | 0.396 | 0.388 | -0.388 |
|  | Time-dependent PS matching | 1.009 | 0.003 | 0.002 | 0.052 | 0.041 | -0.009 |
|  | Longitudinal genetic matching | 1.040 | 0.003 | 0.002 | 0.062 | 0.050 | -0.040 |
| 3: Testing sensitivity of assumed strengths of association between covariates and outcome | Time-invariant PS matching | 1.366 | 0.004 | 0.002 | 0.372 | 0.366 | -0.366 |
|  | Time-dependent PS matching | 1.007 | 0.002 | 0.001 | 0.031 | 0.024 | -0.007 |
|  | Longitudinal genetic matching | 1.041 | 0.001 | 0.001 | 0.048 | 0.042 | -0.041 |

RMSE: root mean squared error; PS: propensity score; SE: standard error; Abs.: absolute

We also considered sensitivity of all matching methods to the following alternate assumed outcomes models:

Base case outcome model:

$y_{it}=z_{it}+\beta_{L}x_{1i}+\beta_{M}x_{2i}+$ $\beta_{H}x_{3i}+$ $\beta_{H}x_{5it}+$ $\beta_{L}x_{6i}+$ $\beta_{M}x_{7i}+$ $\beta_{H}x_{8i}+$ $\beta_{H}x_{10it}+$ $\beta_{L}x_{11it}+$ $\beta_{L}x_{12it}$

(1) Base outcome model with error term where $e=N(0, 0.25)$

$y_{it}=z_{it}+\beta_{L}x_{1i}+\beta_{M}x_{2i}+$ $\beta_{H}x_{3i}+$ $\beta_{H}x_{5it}+$ $\beta_{L}x_{6i}+$ $\beta_{M}x_{7i}+$ $\beta_{H}x_{8i}+$ $\beta_{H}x_{10it}+$ $\beta_{L}x_{11it}+$ $\beta_{L}x_{12it}+e$

(2) Outcome model including additional covariates that previously were only assumed to influence treatment assignment (inclusion of x4 and x9)

$y_{it}= \beta_{0}+z_{it}+ \beta_{L}x_{1i}+ \beta_{M}x_{2i} + \beta_{H}x_{3i} + \beta_{L}x_{4it} + \beta_{H}x_{5it} + \beta_{L}x_{6i} + \beta_{M}x_{7i} + \beta_{H}x_{8i} + \beta_{L}x_{9it} + \beta_{H}x_{10it} + \beta_{H}x_{11it}+\beta_{L}x_{12it}$

(3) Outcome model with altered strengths of association

$$y_{it}= \beta_{0}+z_{it}+ \beta_{H}x_{1i}+ \beta_{L}x_{2i} + \beta_{M}x_{3i} + \beta_{M}x_{4it} + \beta_{M}x_{5it} + \beta_{M}x_{6i} + \beta_{L}x_{7i} + \beta_{H}x_{8i}+ \beta_{L}x_{9it} + \beta_{H}x_{10it} + \beta_{L}x_{11it}+\beta_{L}x_{12it}$$

We find that estimated variance and RMSE were marginally greater in other scenarios compared to the base case. Mean treatment effect estimates and bias remained relatively stable across explored outcome models.

**References**

1. Diamond A, Sekhon JS. Genetic matching for estimating causal effects: A general multivariate matching method for achieving balance in observational studies. *Review of Economics and Statistics.* 2013;95(3):932-945.

2. Lu B. Propensity score matching with time‐dependent covariates. *Biometrics.* 2005;61(3):721-728.

3. Nakahara S, Tomio J, Takahashi H, et al. Evaluation of pre-hospital administration of adrenaline (epinephrine) by emergency medical services for patients with out of hospital cardiac arrest in Japan: controlled propensity matched retrospective cohort study. *Bmj.* 2013;347.

4. Andersen LW, Raymond TT, Berg RA, et al. Association between tracheal intubation during pediatric in-hospital cardiac arrest and survival. *Jama.* 2016;316(17):1786-1797.

5. Sekhon JS. Multivariate and propensity score matching software with automated balance optimization: the matching package for R. *Journal of Statistical Software, Forthcoming.* 2008.
